# Sense of happiness and other aspects of quality of life in patients with obsessive-compulsive disorder

**DOI:** 10.3389/fpsyt.2022.1077337

**Published:** 2022-12-21

**Authors:** Maciej Żerdziński, Marcin Burdzik, Roksana Żmuda, Agnieszka Witkowska-Berek, Paweł Dȩbski, Natalia Flajszok-Macierzyńska, Magdalena Piegza, Hanna John-Ziaja, Piotr Gorczyca

**Affiliations:** ^1^Dr. Krzysztof Czuma’s Psychiatric Center, Psychiatric Department No 2, Katowice, Poland; ^2^Faculty of Medicine, Academy of Silesia, Katowice, Poland; ^3^Institute of Law at Faculty of Law and Administration, University of Silesia in Katowice, Katowice, Poland; ^4^Department of Psychiatry, Faculty of Medical Sciences in Zabrze, Medical University of Silesia in Katowice, Tarnowskie Góry, Poland

**Keywords:** obsessive-compulsive disorder (OCD), quality of life, Oxford Happiness Questionnaire, quality of sexual life, delayed treatment initiation, sense of happiness, wellbeing

## Abstract

**Introduction:**

Quality of life (QoL) is the intellectual and emotional wellbeing of an individual, which is determined by many factors. The most measurable are the sense of happiness, occupational satisfaction, quality of interpersonal relationships and sex life. Obsessive-compulsive disorder (OCD) is a chronic mental disorder diagnosed by the presence of obsessions and compulsions that disrupt normal psychosocial functioning. Despite early onset, treatment is delayed (OCD-DTI) and unsatisfactory.

**Objectives:**

The primary objective of this study is to assess selected correlates of the sense of happiness among patients with OCD. The secondary objective is to compare the sense of happiness with healthy people.

**Methods:**

Seventyfive OCD patients and equal number of healthy subjects were compared using a Polish adaptation of the Oxford Happiness Questionnaire (OHQ-23).

**Results:**

We found a significant negative correlation between sense of happiness and the severity of OCD (*r* = −0.479), the number of obsessive-compulsive personality traits (*r* = −0.323), the number of other comorbid mental disorders (*r* = −0.272), the level of aggression (*r* = −0.448), impulsivity (*r* = −0.301), depressiveness (*r* = −0.357), and the sexual dysfunctions (*r* = −0.279). The latter were much more common in individuals with OCD compared to healthy subjects (66.67 vs. 12%). The level of loneliness was over two times higher in the study group compared to controls (27 vs. 12%). The mean delay in treatment onset was 13 years. Conclusions. Assessment of aspects of QoL should be an integral part of the diagnostic and therapeutic process in OCD.

## 1 Introduction

Quality of life (QoL) is defined by the World Health Organization (WHO) as “individuals’ perceptions of their position in life in the context of the culture and value systems in which they live and in relation to their goals, expectations, standards, and concerns” (1). In medical terms, QoL is the individuals’ intellectual, emotional, and somatic wellbeing, which is defined by a number of factors. The primary ones include satisfaction with life and work, happiness, the ability to build interpersonal relationships; or their absence, which ultimately promotes resignation and suicidal thoughts (2–4). Despite advances in the treatment of mental illnesses, the therapeutic efficacy is still unsatisfactory. Consequently, the remission achieved may be incomplete and the level of wellbeing and ability to happiness impaired (5–8).

Obsessive-compulsive disorder (OCD) is one of conditions that reduce QoL (9). OCD is a chronic mental disorder diagnosed on the basis of the presence of obsessions (persistently recurring, intrusive thoughts, images, impulses) and compulsions (compulsive and transiently satisfying actions performed in response to obsessions) (10). OCD is often associated merely with excess hand washing or checking. In fact, severe and complex OCD rituals, which are referred to as obsessional slowness in extreme cases, prevent normal psychosocial functioning (10–15). The negative impact of obsessions is also experienced by the closest people of the affected person, who helplessly adapt to the patient’s compulsions or respond with either active or passive aggression (12, 16–18). According to various sources, the prevalence of OCD ranges from 1.5 to 3.5%, regardless of gender (19–24). This chronic disorder often has its onset in early childhood and adolescence (25, 26). Thus, a person with chronic OCD may not have experienced psychological normativity *per se* (12). The effectiveness of OCD therapy is most often only partial (70–80%), with 20–30% of patients unresponsive to treatment (27, 28). The short duration of remission and high relapse rates are also not very satisfactory (29). For OCD pharmacotherapy, it is recommended to use significantly higher doses of selective serotonin reuptake inhibitors (SSRI) and clomipramine than those conventionally used in major depressive disorder (MDD) (12, 29, 30). For treatment-resistant OCD, psychosurgical techniques (capsulotomy, cingulotomy) are permitted, which is an extraordinary treatment in the field of psychiatry (31, 32). OCD delayed treatment initiation (OCD-DTI), which is 10 years according to Hollander et al. (33) and up to 13 years according to Ziegler et al. (34) is another problem (33, 34). Obsessions that may extend OCD-DTI include ego-syntonic obsessions and, most often, symmetry obsessions and compulsive ordering and counting (29). Obsessions with absent insight can be misdiagnosed as paranoid delusions. When schizophrenia is diagnosed, a number of further therapeutic misconceptions arise, which can have a negative impact on the wellbeing of the OCD patient (12, 35, 36). It is not uncommon for patients with OCD to see a psychiatrist only because they develop MDD symptoms. Remission of MDD symptoms may be considered a satisfactory treatment outcome, while OCD remains undiagnosed and promotes further MDD relapses (12).

Obsessive-compulsive disorder is known to deteriorate QoL, depending on the presence of various factors (9, 12). In addition to symptoms severity, other important factors include older age, female gender, unemployment, low level of education and poor community support (5, 8, 33, 37–45). Wellbeing in OCD may also be impaired due to comorbidity with other mental disorders. The most common of these include (in random order): MDD, bipolar disorder (BD), anxiety disorder (AD), alcohol use disorder (AUD), and obsessive-compulsive personality disorder (OCPD) (1, 19, 43, 46–48). Another problematic phenomenon of OCD is increased rates of aggression and impulsivity, which are negatively related to various aspects of wellbeing (49, 50). Hollander et al. (33) showed that 73% of OCD patients experience family crises, 62% fail to build close relationships, 58% experience learning problems, 47% experience work problems, and 40% are unfit to work (33). Koran et al. (51) indicated that social functioning of patients with OCD was worse than that of the general population and of diabetes patients (51). Srivastava et al. (52) showed holistic QoL deficits in OCD, even despite achieving partial clinical improvement (52). It is believed that OCD patients not only show deficits in social skills, but are also more likely to experience difficulties in relationships, and thus are less likely to marry (53). Relationship problems are one of the most common causes of sexual dysfunction or poor quality sex (54). Dissatisfaction with sexual life is reported by 54–73% of OCD patients (55–57). Vulink et al. (58) found that women with OCD have reduced or no sex drive, difficulties in achieving an orgasm or an unsatisfactory orgasm, as well as sexual disgust (58). About 47% of patients with OCD do not have a partner or have not had sexual intercourse for years (56).

## 2 Objectives

The aim of this study is to reassess some aspects of QoL in patients with OCD. The primary objective is to assess selected correlates of the sense of happiness (a sense of life satisfaction, a sense of strength, meaning and control over one’s own life) among patients with OCD. The secondary objective is the comparison of the sense of happiness and its ingredients between patients and healthy people. We also verified whether wellbeing in OCD depends on age, gender, severity of symptoms, duration and treatment delay, the quality of sexual life, the number of comorbid mental disorders and OCPD, the severity of depression, mania, aggression and impulsivity.

## 3 Materials and methods

The study group included 75 patients diagnosed with and treated for OCD. The primary inclusion criterion was a diagnosis of OCD. To avoid bias, we decided to select the majority of patients without sudden OCD exacerbation and without severe mood disorders. For this reason, the vast majority of patients were randomly selected from our outpatient department (*N* = 66). A smaller number (*N* = 9) were treated in our psychiatric unit (for follow-up). The control group consisted of 75 individuals without mental disorders. Volunteers were selected from among medical personnel and then from among their colleagues (snowball method). We did not use media advertising in the study. The exclusion criterion for the healthy group was the presence of a mental disorder (including OCD). In the control group, the significant variables were to find volunteers similar in age and gender to group 1. None of the healthy volunteers were associated with the OCD group. Both groups formed naturally (by random chance) in terms of income, habitat and culture. Group 1 was assessed for correlations between various aspects of wellbeing: sense of happiness, life satisfaction, sense of strength, sense of perceived and control over one’s own life, quality of sexual life and age, gender, the severity of OCD symptoms, the total number of comorbid mental disorders, presence of OCPD as well as the level of depression, mania, aggression and impulsivity. We also assessed treatment duration and delay in group 1. Then we compared the study group with the control group for a sense of happiness (life satisfaction, sense of strength, sense of perceived, and control over one’s own life), and quality of sexual life.

The study was conducted after informed consent was obtained from each patient, having been explained about the purpose, nature and procedure of the study. The study caused no additional burden for the patients: apart from its diagnostic value, it was also psychoeducational in nature. The local bioethics committee has approved the study. All investigators were medical professionals (psychiatrists, psychologists, and psychiatric nurse) employed in center that diagnose and treat mental disorders, with extensive experience in the diagnosis and therapy of OCD. Each of the investigators was trained for all the diagnostic tools used. The obtained results were each time assessed simultaneously by at least two researchers to ensure the reliability of the research. None of the patients had any problems with defining their gender identity. The following diagnostic tools were used:

1. Yale*-*Brown Obsessive Compulsive Scale (Y-BOCS). The tool is used to measure OCD severity and to monitor treatment outcomes. It measures five OCD parameters (time occupied, associated distress, impairment, resistance, and control of obsessions and compulsions). Total score indicates OCD severity as subclinical (0–7), mild (8–15), moderate (16–23), severe (24–31) and extremely severe (32–40) (59).
2. The Oxford Happiness Questionnaire; Polish version (OHQ-23). The tool is used to assess global mental wellbeing in the following dimensions: a sense of happiness (life satisfaction, sense of strength, sense of perceived, and control over one’s own life). The minimum and maximum total score is 23 and 138, respectively. OHQ-I (life satisfaction and sense of strength) consists of 14 statements; the minimum and maximum score is 14 and 84, respectively. OHQ-II (sense of meaning and control) consists of nine statements; the minimum and maximum score is 9 and 54, respectively (60).
3. Arizona Sexual Experiences Scale (ASEX). This screening tool consists of five items quantifying sex drive, arousal, vaginal lubrication/penile erection, ability to reach orgasm, and satisfaction from orgasm (rated from 1 to 6; with a total score of 5 to 30). A high total score (≥19) indicates the presence of sexual disorders (61).
4. Hamilton Depression-Rating Scale (HDRS-17). This multi-element questionnaire is used for the diagnosis of depressive disorders and the assessment of recovery. The answers are rated from 0 to 4. The total score indicates the severity of depression (<7–no depression; 8–16–mild depression, 17–23–moderate depression; 18 and 29–severe depression, ≥30–very severe depression) (62).
5. Young Mania Rating Scale (YMRS) is an 11-item tool to assess manic symptoms. The answers are rated on a 5-point scale (0–4), except for 4 items, which are scored 0–8. The total score of ≥ 29 is considered the cut-off point for severe mania (63).
6. Hypomania Check List (HCL-32). The tool investigates the presence of hypomanic and manic symptoms. HCL-32 is a self-assessment questionnaire consisting of 7 parts. It assesses mood, activity, and drive. A score of ≥ 14 is the cut-off point that may indicate hypomania or mania (64).
7. Buss-Perry Aggression Questionnaire (BPAQ). This 29-item tool is used to assess the general level of aggression and its components: physical aggression (PA)–9 items; verbal aggression (VA)–5 items; anger (A)–7 items; hostility (H)–8 items (65).
8. Barratt Impulsiveness Scale (BIS-11) is a 30-item tool to measure the three theoretical subtraits of impulsiveness; i.e.,–cognitive, motor, and non-planning impulsiveness (66).
9. The total number of comorbid mental disorders (OCD-Com). The prevalence (the order is random) was assessed for the most common comorbidities of OCD: MDD, BD, AD and AUD. It was assumed that only the total number of comorbid mental disorders would be relevant to the study (0 to 4). All these disorders were diagnosed retrospectively based on medical history and treatment records provided, verified in accordance with the DSM 5 criteria by psychiatrists (10).
10. The occurrence of OCPD was assessed separately (according to DSM 5 criteria verified by psychiatrists), as it was assumed to be conducive to ego-syntonic obsessions (which can reduce quality of life, as described in the introduction). The survey was based on questions correlating with obsessive-compulsive personality traits. Four or more traits (out of a total of eight) were considered significant for diagnosis. (10).
11. Questions regarding duration and delay of OCD treatment were diagnosed retrospectively based on medical history and treatment records provided.

Excel 2016 and Statistica version 13.3 were used for data processing. The normality of the distribution of variables was verified with the Shapiro–Wilk test. The correlation matrix was prepared using the Spearman rank correlation coefficient. The Mann–Whitney *U* test was used to test the significance of differences between the group of patients and healthy controls. The level of statistical significance was set at α ≤ 0.05.

## 4 Results

The description of the study and control group in terms of age, gender, education, occupational activity, and interpersonal relations is presented in Table 1. Basic descriptive statistics of variables in the study group are presented in Table 2.

**TABLE 1:** Basic parameters in study vs. control group.

	Study group (*N* = 75)	Control group (*N* = 75)
Gender [%]	♀ = 53.33%; ♂ = 46.67%	♀ = 60%; ♂ = 40%
Mean age [years]	45.12	43.55
Age _Max/Min_ [years]	73/24	66/26
**Education**		
Higher	58.67%	57.33%
Secondary	29.33%	18.67%
Vocational	10.67%	8%
Elementary	1.33%	16%
**Occupational status**		
Employed	61.33%	89.33%
Disability pension due to OCD	26.67%	0%
Disability pension for other reasons	1.33%	0%
Retirement pension	9.33%	9.33%
Unemployed (no pension)	5.33%	2.67%
**Interpersonal relationships**		
Formal relationship	46.67%	57.33%
Informal relationship	17.33%	30.67%
Single	27%	12%

**TABLE 2:** Descriptive statistics of variables in the obsessive-compulsive disorder (OCD) group.

Variable	Mean	SD	Median	CI–95%	CI + 95%
OCD	22.627	11.150	22.000	9.607	13.288
Com	1.507	0.828	2.000	0.714	0.987
Y-BOCS	22.787	7.161	24.000	6.170	8.534
OCPD	3.880	2.175	4.000	1.874	2.592
B-P S	78.827	16.968	80.000	14.620	20.221
Imp	63.467	10.965	62.000	9.447	13.067
HCL	0.547	0.501	1.000	0.432	0.597
YMRS	3.773	4.675	2.000	4.028	5.571
HDRS	8.747	6.147	8.000	5.296	7.326
ASEX	20.067	6.761	19.000	5.825	8.057
OHQ	85.440	20.206	86.000	17.410	24.080
OHQ I	53.813	13,668	55.000	11.776	16.288
OHQ II	31.627	9.171	33.000	7.902	10.929

OCD, duration of OCD; Com, number of comorbid mental disorders; Y-BOCS, general severity of OCD; OCPD, number of anankastic personality traits; B-Pe S, Buss-Perry Scale; Imp, general intensity of impulsivity; HCL, the intensity of hypomania; YMRS, Young Mania Rating Scale; HDRS, Hamilton Depression Scale; ASEX, Arizona Sexual Experience Scale; OHQ, Oxford Happiness Questionnaire; OHQ I, life satisfaction and a sense of strength; OHQ II, sense of meaning and control.

The analysis of correlations between the variables in the OCD group showed statistically significant relationships. Happiness (OHQ) significantly and negatively correlated with the number of comorbid mental disorders (Com, *r* = −0.272). A negative relationship was also found between the OHQ of patients with selected psychopathological phenomena such as the severity of OCD symptoms (Y-BOCS, *r* = −0.479), number of OCPD traits (*r* = −0.323), the severity of aggression (B-PS, *r* = −0.448) and impulsivity (Imp, *r* = −0.301) and the severity of depression (HDRS, *r* = −0.357) and sexual dysfunctions (ASEX, *r* = −0.279). As for life satisfaction and sense of strength (OHQ I), significant negative correlations were observed with the severity of compulsions (*r* = −0.449), aggression (*r* = −0.482) and impulsivity (*r* = −0.311), as well as with the severity of depressive symptoms (*r* = −0.366) and sexual dysfunctions (*r* = −0.249). Meanwhile, the sense of meaning and control (OHQ II) showed significant and negative correlations with the number of comorbid mental disorders (*r* = −0.360), the severity of OCD symptoms (*r* = −0.391), the number of OCPD traits (*r* = −0.372), aggression (*r* = −0.282), severity of depressive symptoms (*r* = −0.294), and the severity of sexual dysfunctions (*r* = −0.239). All correlations were statistically significant at *p* ≤ 0.05 (Table 3).

**TABLE 3:** Correlations between happiness and selected psychopathological phenomena in the obsessive-compulsive disorder (OCD) group.

	OCD	Com	Y-BOCS	OCPD	B-P S	Imp	HCL	YMRS	HDRS	ASEX	OHQ	OHQ I	OHQ II
OCD	1.000	0.274*	0.301*	0.441*	0.050	0.201	0.070	0.066	0.213	0.265*	-0.156	-0.083	-0.147
Com		1.000	0.463*	0.359*	0.132	0.157	-0.127	0.189	0.367*	0.340*	-0.272*	-0.173	-0.360*
Y-BOCS			1.000	0.395*	0.200	0.308*	0.234*	0.342*	0.414*	0.229*	-0.479*	-0.449*	-0.391*
OCPD				1.000	0.252*	0.125	0.402*	0.316*	0.257*	0.253*	-0.323*	-0.226	-0.372*
B-P S					1.000	0.369*	0.176	0.178	0.215	0.033	-0.448*	-0.482*	-0.282*
Imp						1.000	0.194	0,091	0.078	0.065	-0.301*	-0.311*	-0.187
HCL							1.000	0.331*	0.040	0.035	-0.048	0.029	-0.162
YMRS								1.000	0.083	0.020	-0.054	-0.062	-0.021
HDRS									1.000	0.101	-0.357*	-0.366*	-0.294*
ASEX										1.000	-0.279*	-0.249*	-0.239*
OHQ											1.000	0.907*	0.800*
OHQ I												1.000	0.515*
OHQ II													1.000

OCD, duration of OCD; Com, number of comorbid mental disorders; Y-BOCS, general severity of OCD; OCPD, number of anankastic personality traits; B-Pe S, Buss-Perry Scale; Imp, general intensity of impulsivity; HCL, the intensity of hypomania; YMRS, Young Mania Rating Scale; HDRS, Hamilton Depression Scale; ASEX, Arizona Sexual Experience Scale; OHQ, Oxford Happiness Questionnaire; OHQ I, life satisfaction and a sense of strength; OHQ II, sense of meaning and control, *p ≤ 0.05.

The results for the study group in terms of the number of comorbidities are shown in Table 4.

**TABLE 4:** Number of comorbidities in patients with obsessive-compulsive disorder (OCD).

OCD-Com	MDD	AD	BD	AUD
Patients (*N* = 75)	36	48	21	8
OCD-Com = 0	9			
OCD-Com = 1	26			
OCD-Com = 2	33			
OCD-Com = 3	7			
OCD-Com = 4	0			

OCD-Com, number of obsessive-compulsive comorbidities; MDD, major depressive disorder; AD, anxiety disorders; BD, bipolar disorder; AUD, alcohol use disorder.

**FIGURE 1:**
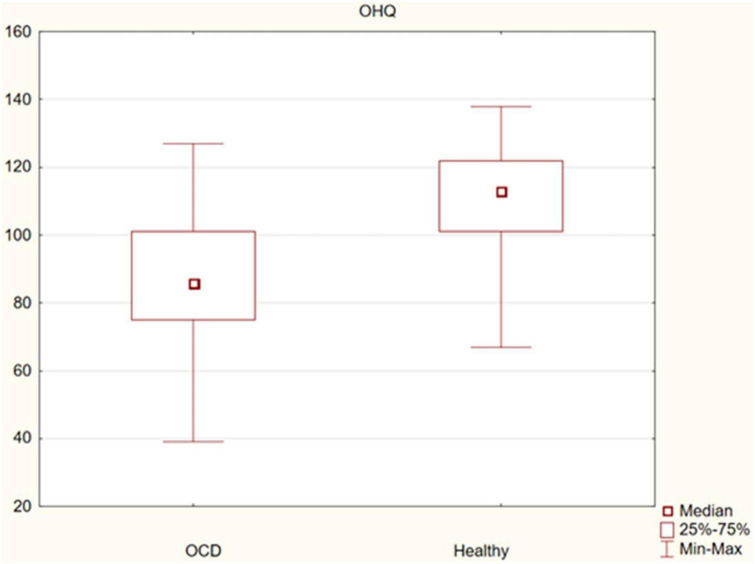
Differences in happiness between obsessive-compulsive disorder (OCD) patients and healthy controls.

**FIGURE 2:**
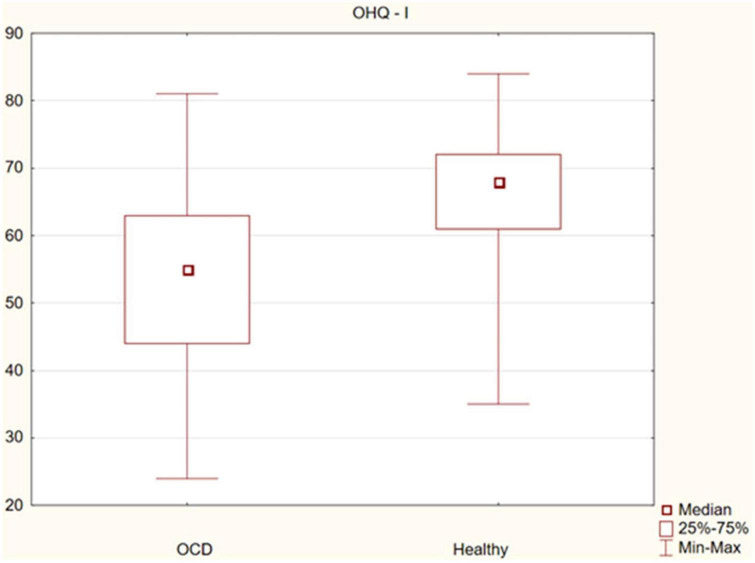
Differences in life satisfaction and sense of strength between obsessive-compulsive disorder (OCD) patients and healthy controls.

**FIGURE 3:**
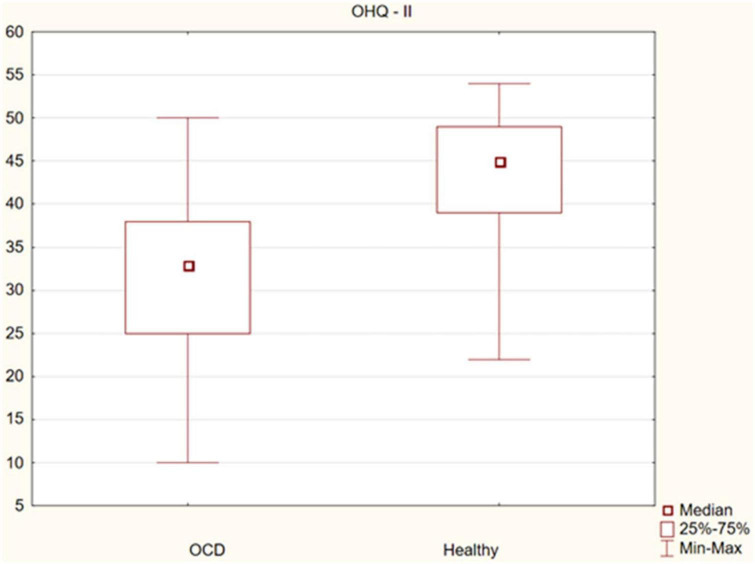
Differences in the sense of life meaning and perceived control between obsessive-compulsive disorder (OCD) patients and healthy controls.

The assessment of the significance of differences in happiness between OCD patients and healthy controls showed statistically significant differences in the sense of happiness (median 86 vs. 113, *p* = 0.000), as well as its subscales: life satisfaction, sense of strength (median 55 vs. 68, *p* = 0.000) and sense of meaning and control (median 33 vs. 45, *p* = 0.000). Happiness was found to be significantly lower in the OCD group. The observed differences are presented in Figures 1–3.

Sexual dysfunctions (SD) were found in 66.67% of the OCD respondents (mean = 20.067, SD = 6.761) and 12% of healthy controls (mean = 13.973, SD = 4.090). They were more common among women (77.5%, mean = 21.625, SD = 6.686) than men (54.29%, mean = 18.286, SD = 6.488). Women most often reported lack of pleasure from orgasm (54.84%), complete inability to achieve orgasm (51.61%), and vaginal dryness during intercourse (41.94%). Complete failure to achieve orgasm (52.63%) and inability to achieve erection (36.84%) were the most common dysfunctions reported by men.

No significant correlation was found between age and any of the happiness variables: (OHQ: *r* = 0.003; OHQ-I: *r* = 0.042; OHQ-II *r* = −0.061). Gender also did not significantly differentiate any of the OHQ parameters (OHQ: *p* = 0.638; OHQ-I: *p* = 0.944; OHQ-II: *p* = 0.749).

The mean OCD treatment delay was 13 years (from <1 year to 44 years). The analysis of correlations between the variables in the OCD group revealed no statistically significant relationships for the OCD-DTI parameter.

## 5 Discussion

It is difficult to explore all dimensions of the quality of life in OCD patients. Theoretically, their number may be infinite, and therefore immeasurable. In this study, two factors determined the final selection of the predictors investigated. First, the severity of symptoms that are commonly considered to cause discomfort (OCD symptoms, depression, aggression, impulsivity, comorbidity) was assessed. Secondly, factors commonly considered important for existential wellbeing (relationship, sexual, and occupational spheres) were analyzed. Happiness measured with The Oxford Happiness Questionnaire (the OHQ-23 version) was the leading predictor of QoL.

Our study confirmed significant wellbeing deficits in OCD patients compared to healthy individuals. This was found for all studied dimensions: subjective happiness, perceived control over one’s own life, problems in interpersonal relationships, as well as sexual and occupational life. It is worth noting that the percentage of people who did not have relationships in the study group was over two times higher than in the control group (27 vs. 12%). It should be assumed that loneliness was not a conscious choice of the affected person, but a consequence of OCD.

Almost 67% of respondents reported problems in their sexual life, which confirms the results of previous studies in this area (54–73%) and the cited thesis that problems in relationships are one of the most common causes of poor quality sex (54–57). It is therefore not surprising that the negative impact of sexual disorders on happiness, life satisfaction, sense of strength, meaning and control has been proven. Sexual dysfunctions were much more common in women than in men (66.67 vs. 54.29%; mean). Undoubtedly, from a holistic point of view, sexual dysfunction is common in the course of OCD, which further reduces not only the sense of happiness, but many other aspects of an individual’s QoL. It would be worth exploring this issue in further and more in-depth studies.

Obsessive-compulsive disorder resulted in occupational disability in over 25% of respondents. This result is definitely unfavorable, especially since until recently, OCD was considered a neurotic disorder (obsessive compulsive neurosis) (67). Considering a 1996 study by Hollander (40% of those with occupational disability), it is also a much more positive result and, with a high degree of caution, it may be considered to indicate a measurable progress in OCD therapeutic efficacy.

The strongest and most detrimental effect on ability to happiness was noted for the severity of OCD. This confirms the disastrous psychosocial consequences of the intensification of OCD symptoms and requires no further interpretation.

We have also shown that OCD is often accompanied by other mental disorders. The mean number of comorbidities in the study group is 1.35 Com; most often, two (44% responders) and one (34,6% respondents). The co-existence of three additional psychiatric disorders was found in 9.33% of the subjects, and none of the patients had four comorbidities. Our study did not verify whether it was co-morbidity, co-incidence, or co-occurrence. It was important to demonstrate this phenomenon, which should be assumed to have contributed to reduced happiness, especially through the investigated negative impact on the sense of meaning and control over one’s own life (OHQ-II). Further research is needed to assess this topic in more detail.

We also confirmed the disruptive effect of OCPD on all measured aspects of wellbeing. This finding indirectly corresponds to the study cited in the introduction, which indicated that ego-syntonic obsessions (problematic in terms of self-identification and thus insight) have a particularly negative impact on QoL (29). It is worth mentioning that the phenomenon of OCPD in the course of OCD is often marginalized in clinical practice, while its presence intensifies compulsivity *per se*, and increases disease severity, ultimately increasing treatment resistance (12).

Following up on previously cited studies, it was found that a significant problem in patients with OCD is a increase in symptoms of depression, aggression and impulsivity. It remains a rhetorical question and further research is needed to investigate on whether they are a consequence or a psychological component of OCD. Regardless of the etiological uncertainties, all these factors negatively correlated with happiness, life satisfaction, sense of strength, meaning and control (OHQ). The presence of depression, aggression and impulsivity certainly makes it difficult (or impossible) to maintain interpersonal or professional relationships, as confirmed by the results of our study. Thus, it can reduce the holistic aspects of a patient’s quality of life.

The study found that the mean treatment delay was up to 13 years. Although no significant association was found between OCD-DTI and all aspects of wellbeing studied, this result encourages further clinical reflection. It is worse compared to the results obtained many years ago by Hollander et al. (33) and similar to Ziegler et al. (34). It may mean that despite the unquestionably improved access to medical knowledge and psychiatric treatment, OCD is still an embarrassing and hidden disorder. The results of our study did not answer the question about the cause of this phenomenon. It can be assumed that OCD symptoms are already perceived indiscriminately (ego-syntonically) or considered too intimate to be exhibited. They can also be specifically related to psychological (seemingly protective) functions (12). In this sense, significant OCD-DTI may not only indirectly affect the QoL, but also signal problematic insight. Further research is needed to assess this topic in more detail.

The study did not confirm that the QoL in OCD patients depends on age or gender. When analyzing the lack of such relationships, it should be taken into account that the vast majority of elderly respondents (regardless of gender) sought long-term psychiatric treatment, which could have contributed to improved QoL.

## 6 Limitations of the study

Certain limitations of this study should be noted. First of all, the study investigated only selected (most transparent) aspects of QoL in patients with OCD, which means that there are still some areas of wellbeing that require further exploration. The relatively small study sample made it difficult to generalize the obtained results and relate them to the general population of OCD patients. Some limitations result from difficulties in objectivizing the data from the interview, especially in terms of embarrassing information, e.g., data on sexual dysfunctions, which may be the reason for their non-disclosure by some patients, both in the study and the control group. At this point it should be noted that ASEX tests were mostly conducted by women, which could induce male patients to underestimate their sexual problems. Adverse effects of commonly used OCD medications (SSRI), which may reduce sexual satisfaction, should also be considered. The high percentage of comorbidities in the study group makes it difficult to draw conclusions about the sense of happiness of patients with isolated OCD. It should be noted that a limited number of comorbidities were investigated, and their course was not taken into account. The problem of comorbidity, due to its extensiveness, will be analyzed in detail in another study. OCD delay–the result may be confounded due to a small group of patients with OCD. The two groups investigated were matched only in terms of age and gender, while the other variables (education, professional and financial status, interpersonal relationships) were different. This may have made it difficult to compare them and affected the strength of the correlations reported in the results.

## 7 Conclusion

1. Obsessive-compulsive disorder reduces ability to happiness and other aspects of QoL.
2. Loneliness, existential and sexual frustration are psychosocial consequences of OCD.
3. In addition to symptoms severity, depression, aggression, and impulsivity are factors that destabilize wellbeing in OCD. These are not included in common definitions of OCD. Therefore, they are worth considering and treating them.
4. Other mental disorders should be co-diagnosed and co-treated to improve all aspects of QoL in OCD.
5. The diagnosis of OCD is severely delayed.
6. Assessment of specific aspects of QoL–especially a sense of happiness–should be an integral part of the diagnostic and therapeutic process in OCD.

## Data availability statement

The raw data supporting the conclusions of this article will be made available by the authors, without undue reservation.

## Ethics statement

The studies involving human participants were reviewed and approved by the Bioethics Committee at the Silesian Medical Chamber. The patients/participants provided their written informed consent to participate in this study.

## Author contributions

MŻ: development of research aims, scope, methodology, collection and analysis of research data, literature review, and creation of the main text (main author). MB: development of research aims and methodology, collection and analysis of research data, literature review, and co-authorship of the main text. RŻ: collection and analysis of research data, preparation of text fragments, and literature review. AW-B: collection and analysis of research data, preparation of text fragments, development of research aims and methodology, and literature review. PD: statistical analysis of research data, preparation of text fragments, and verification of methodological assumptions. NF-M: collection and analysis of research data and literature review. MP and HJ-Z: collection and analysis of research data and verification of methodological assumptions. PG: collection and analysis of research data, verification of methodological assumptions, and aims of the study. All authors contributed to the article and approved the submitted version.
